# Genotype × environment interaction: trade-offs between the agronomic performance and stability of durum (*Triticum turgidum*) wheat to stem-rust resistance in Kenya

**DOI:** 10.3389/fpls.2024.1427483

**Published:** 2024-07-25

**Authors:** Emmaculate A. Ogutu, Sammy L. Madahana, Sridhar Bhavani, Godwin Macharia

**Affiliations:** ^1^ Kenya Agricultural and Livestock Research Organization (KALRO), Njoro, Kenya; ^2^ World Agroforestry Centre (ICRAF House), International Maize and Wheat Improvement Center (CIMMYT), Nairobi, Kenya; ^3^ International Maize and Wheat Improvement Center (CIMMYT), Texcoco, Mexico

**Keywords:** durum wheat, stem rust, genotype-environment interaction, genetic variation, agronomic performance

## Abstract

Stem rust significantly threatens durum wheat production, often resulting in substantial yield losses. To better understand resistance mechanisms and the stability of durum lines in stem rust-prone environments, this study evaluated 49 durum genotypes over three seasons at the Kenya Agricultural and Livestock Research Organization in Njoro. Utilizing 7 × 7 alpha lattice design, we assessed adult-plant resistance, monitored disease progression through final disease score (FDS) and area under the disease progress curve (AUDPC), and evaluated agronomic performance. Statistical analyses revealed significant seasonal and genotypic effects on FDS, AUDPC, spike length, and grain yield (p≤0.01; p≤0.001), with important genotype-by-season interactions (p≤0.05; p≤0.001). Broad-sense heritability for AUDPC was high at 0.91 and moderate at 0.35 for kernels per spike, underscoring the genetic basis of these traits. Notably, we observed negative correlations between disease parameters and agronomic traits, suggesting potential trade-offs. GGE biplot analysis singled out the first season (main season of 2019) as crucial for evaluating stem rust resistance and identified several durum lines, such as G45 and G48, as consistently resistant across all conditions. Furthermore, this analysis highlighted G45, G48, G176 and G189 as the highest yielding and most stable lines. The discovery of these resistant and high-performing genotypes is critical for enhancing durum breeding programs, helping to mitigate the impact of stem rust and improve yield stability.

## 1 Introduction

Durum wheat (Triticum turgidum, 2n = 4x = 28, AABB), a staple tetraploid wheat species native to Sub-Saharan Africa, is an important crop in global agriculture, producing approximately 36 million tons per year (Biggeri et al., 2018; Tidiane Sall et al., 2019). Despite its agricultural importance, durum wheat production is frequently hampered by combination of biotic and abiotic stressors, such as drought and heat, diseases like rust (*Puccinia* spp.), and poor farming practices (Fahad et al., 2017; Kalender and Dogan, 2021; Seleiman et al., 2021). Among these, stem rust caused by *Puccinia graminis* f.sp. *tritici* (*Pgt*) is particularly devastating (Figueroa et al., 2016, 2018, 2023), causing severe crop losses in major growing regions such as Kenya and exacerbating food insecurity (Singh et al., 2015; Fetch et al., 2021; Wanyera and Wamalwa, 2022). The goal of this study is to investigate novel breeding strategies for increasing stem rust resistance in durum wheat, thereby improving yield stability and food security.

Recurrent stem rust epidemics have resulted in large-scale production losses (Singh et al., 2015), posing serious and imminent threat to food and nutrition security in Kenya and other durum wheat production regions. While durum adoption in Kenya lags behind Ethiopia, food insecurity urges diversification with durum as key cereal because of increasing consumption of durum products. The emergence of race TTKSK (*Ug99*) in Africa has rendered the widely used stem rust resistance gene *Sr31* (Zhang et al., 2017) and multiple additional *Sr* genes ineffective (Pretorius et al., 2000). It was anticipated that this race would continue to spread to Iran, South Africa, Yemen, and East Africa (Singh et al., 2015; Patpour et al., 2020).

The global threat posed by *Ug99* stem rust strain and its variants, which have compromised previously effective resistance genes such as *Sr31*, highlights the critical need for ongoing genetic innovation (Pretorius et al., 2000; Singh et al., 2015). Over 13 virulent variants of *Ug99* have been identified, exposing up to 95% of global wheat cultivars to stem rust (Newcomb et al., 2016; Mergessa et al., 2020; Bhavani et al., 2022). The rapid evolution of these pathogens emphasizes the importance of a proactive approach in breeding programs that incorporates a broader base of resistance genes from both tetraploid and hexaploid wheat varieties (McIntosh et al., 1995; Park, 2015).

Novel resistance genes are being incorporated into durum wheat breeding programs to address global imperative, which goes beyond regional concerns. Since *Puccinia graminis* f. sp. *tritici* (*Pgt*) does not consider national borders, stem rust poses persistent threat to food security worldwide. Reducing the reliance on fungicides globally is essential for lowering production costs and boosting the economic resilience of farming communities across the globe. This can be achieved through strategic integration of these resistance genes into breeding programs (Singh et al., 2011; Park, 2008). We focused on comprehensive phenotypic analysis of durum wheat genotypes in order to discover and define these novel sources of resistance. This method offers useful foundation for selection in breeding programs by enabling the direct observation of resistance under various environmental conditions (Chao et al., 2017; Bhavani et al., 2022). Our focus was to identify durum wheat cultivars that demonstrate durable resistance to both current and emerging *Pgt* races by fusing conventional breeding techniques with cutting-edge genomic technologies. In light of the danger posed by emerging infections, such initiatives are crucial for maintaining wheat output and enhancing yield stability (Chaves et al., 2013; Singh et al., 2016). This approach is in line with sustainable farming techniques, which are essential for ensuring future food security, and it also lessens the effects of deadly diseases like stem rust (Ellis et al., 2014; Nirmala et al., 2017).

Collaboratively, this effort is being carried out collaboratively with international organizations such as CIMMYT, the Kenya Agriculture and Livestock Research Organization, and the Ethiopia Institute of Agricultural Research. These collaborations have resulted in over 500 new durum wheat varieties adapted to wide range of environmental conditions, demonstrating the power of collaborative research and development (Macharia and Ngina, 2017; Bhavani et al., 2019). Furthermore, our understanding of genotype-environment interactions has revolutionized with the application of complex statistical models such as the Additive Main Effects and Multiplicative Interaction (AMMI) and the Genotype Main Effect (Yan and Tinker, 2006) and Genotype by Environment Interaction (GGE) biplots (Gauch, 2006, 2013). These models have improved our capacity to choose genotypes that reliably yield high and stable yields under various climatic conditions (Mohammadi and Amri, 2013; Heidari et al., 2016; Kendal et al., 2019).

In addition to providing important new insights into the genetic underpinnings of stem rust resistance, this research aimed to support the sustainability of durum wheat production worldwide, promising food security in the face of changing biotic threats. Through series of comprehensive field trials conducted in Njoro, Kenya, and analysis of CIMMYT’s large durum germplasm pool, we hoped to discover new genetic resources that would strengthen the regional and global’s durum wheat supply in the face of stem rust’s persistent challenge.

## 2 Materials and methods

### 2.1 Experimental site

**Figure 1:**
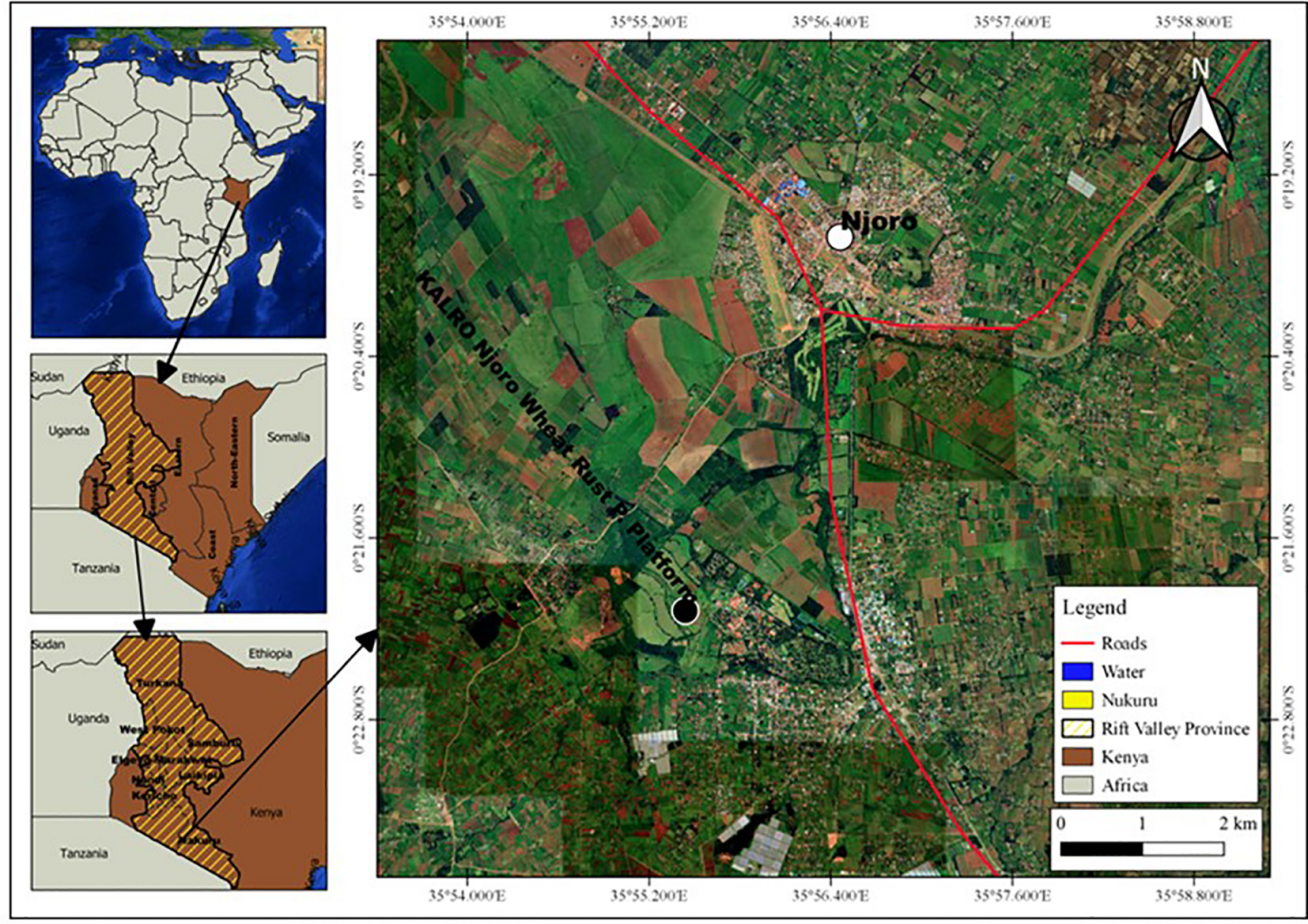
A schematic map is presented,depicting the specific area in Kenya where the experiment was conducted: Kenya: 1.2921° S latitude; 36.8219° E longitude,Nakuru: 0.3030° S latitude; 36.0800° E longitude,Njoro: 0.2004° S latitude; 35.9381° E longitude,KALRO (Kenya Agricultural and Livestock Research Organization) Njoro Wheat Rust Phenotyping Platform: 0.20513° S latitude; 35.56801° E longitude.

**Figure 2:**
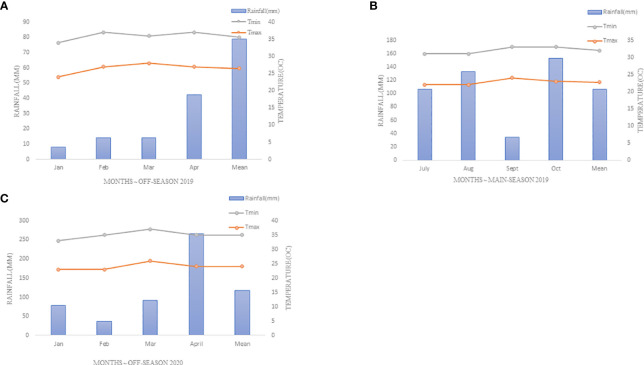
A weather graph presented,depicting specific rainfall (mm),minimum and maximum temperatures (0C) during the experiment period at Njoro-Kenya 2019-2020 **(A)** Season One (Off-Season (OS) 2019), **(B)** Season Two (Off-Season (OS) 2019-2020), **(C)** Season Three (Main-Season (MS) 2020).

The experiment was conducted at Kenya Agricultural and Livestock Research Organization (KALRO), Njoro, for three seasons, *i.e* off-season 2019 (Season 1), main-season 2019 (Season 2) and off-season 2020 (Season 3). The site is located at 35° 55′ 60″ E, 0° 19′ 60″ S with an elevation of 2185 m above sea level in lower highland agro-ecological zone III (LH3) with predominant well-drained mollic andosols soils. The specific location was at KALRO wheat rust phenotyping platform, which is situated at 0.20513°S latitude; 35.56801°E longitude and 2171m elevation, as shown in **Figure 1**. The mean annual precipitation is 1000mm with minimum and maximum temperature of 9°C and 22°C respectively (KALRO Meteorological Station No. 903502) (Shewry, 2009; Shewry and Hey, 2015). The weather conditions experienced during the growing period are as shown in **Figure 2**.

### 2.2 Plant materials

A set of 49 durum genotypes derived from CIMMYT collection lines along with stem rust susceptible checks (Cacuke and PBW343) were evaluated as represented in **Table 1**. They were selected based on stem rust severity, morphology (height, spike and uniformity in particular), grain yield and biomass production.

**Table 1:** Pedigree crosses of selected durum germplasm used in the experiment.

Name/Pedigree Information	Ent. Sel	Accession Number	Synonym	*Species*	Habit	Locality
CIRNO C 2008/4/SOOTY_9/RASCON_37//JUPARE C 2001/3/SOOTY_9/RASCON_37//CAMAYO	30	584003	181003	*Triticum turgidum*	Spring	CIMMYT
PLATA_7/ILBOR_1//SOMAT_3/7/CHEN_11/POC//TANTLO/5/ENTE/MEXI_2//HUI/4/YAV_1/3/	41	573163	181016	*Triticum turgidum*	Spring	CIMMYT
BRONTE/4/ARMENT//SRN_3/NIGRIS_4/3/CANELO_9.1/10/RCOL/THKNEE_2/9/USDA595/3/D67.3/	45	584306	181020	*Triticum turgidum*	Spring	CIMMYT
OROBEL//BUSHEN_4/2*GREEN_18/8/GEDIZ/FGO//GTA/3/SRN_1/4/TOTUS/5/ENTE/MEXI_2//HUI/4/	48	584355	181023	*Triticum turgidum*	Spring	CIMMYT
P91.272.3.1/3*MEXI75//2*JUPARE C 2001/5/ARTICO/AJAIA_3//HUALITA/3/FULVOUS_1/MFOWL_13/4/	49	584369	181024	*Triticum turgidum*	Spring	CIMMYT
SOOTY_9/RASCON_37//SOMAT_3.1/3/SOOTY_9/RASCON_37//STORLOM/4/SOOTY_9/RASCON_37//	68	607973	182009	*Triticum turgidum*	Spring	CIMMYT
CBC 509 CHILE/6/ECO/CMH76A.722//BIT/3/ALTAR 84/4/AJAIA_2/5/KJOVE_1/7/AJAIA_12/	108	608002	182059	*Triticum turgidum*	Spring	CIMMYT
P91.272.3.1/3*MEXI75//2*JUPARE C 2001/11/BOOMER_33/ZAR/3/BRAK_2/AJAIA_2//SOLGA_8/10/	176	607489	182153	*Triticum turgidum*	Spring	CIMMYT
SOOTY_9/RASCON_37//JUPARE C 2001/5/GREEN/SOMO/3/GODRIN/GUTROS//DUKEM/4/YAZI_1/	247	607544	182262	*Triticum turgidum*	Spring	CIMMYT
INRAT 102/11/E90040/MFOWL_13//LOTAIL_6/3/PROZANA/ARLIN//MUSK_6/9/USDA595/3/D67.3/RABI//	411	607820	182630	*Triticum turgidum*	Spring	CIMMYT
JANDAROI/6/YAZI_1/AKAKI_4//SOMAT_3/3/AUK/GUIL//GREEN/5/2*NETTA_4/DUKEM_12//RASCON_19/3/	465	607678	182728	*Triticum turgidum*	Spring	CIMMYT
WOLLAROI/12/LABUD/NIGRIS_3//GAN/3/AJAIA_13/YAZI/10/PLATA_10/6/MQUE/4/USDA573//QFN/	487	607953	182765	*Triticum turgidum*	Spring	CIMMYT
SIMETO/3/SORA/2*PLATA_12//SRN_3/NIGRIS_4/5/TOSKA_26/RASCON_37//SNITAN/4/ARMENT//	569	584299	10583	*Triticum turgidum*	Spring	CIMMYT
BRONTE/4/ARMENT//SRN_3/NIGRIS_4/3/CANELO_9.1/10/RCOL/THKNEE_2/9/USDA595/3/D67.3/RABI//	570	584306	10587	*Triticum turgidum*	Spring	CIMMYT
1A.1D 5 + 1-06/3*MOJO//RCOL/3/SNITAN/SOMAT_3//FULVOUS_1/MFOWL_13/10/AVILLO_1/3/CANELO_8//	571	584313	10590	*Triticum turgidum*	Spring	CIMMYT
B04-17/7/ZENIT/5/SORA/2*PLATA_12//RASCON_37/4/ARMENT//SRN_3/NIGRIS_4/3/CANELO_9.1/6/	1168	665287	700738	*Triticum turgidum*	Spring	CIMMYT
B04-17/7/ZENIT/5/SORA/2*PLATA_12//RASCON_37/4/ARMENT//SRN_3/NIGRIS_4/3/CANELO_9.1/6/	1170	665287	700740	*Triticum turgidum*	Spring	CIMMYT
CAPAROI/6/ALAMO: DR/4/ARMENT//SRN_3/NIGRIS_4/3/CANELO_9.1/5/PLATA_6/GREEN_17//	1171	665294	700741	*Triticum turgidum*	Spring	CIMMYT
HYPERNO/6/YAZI_1/AKAKI_4//SOMAT_3/3/AUK/GUIL//GREEN/5/2*NETTA_4/DUKEM_12//	1172	665296	700742	*Triticum turgidum*	Spring	CIMMYT
HYPERNO/6/YAZI_1/AKAKI_4//SOMAT_3/3/AUK/GUIL//GREEN/5/2*NETTA_4/DUKEM_12//	1173	665296	700743	*Triticum turgidum*	Spring	CIMMYT
HYPERNO/6/YAZI_1/AKAKI_4//SOMAT_3/3/AUK/GUIL//GREEN/5/2*NETTA_4/DUKEM_12//	1174	665296	700744	*Triticum turgidum*	Spring	CIMMYT
HYPERNO/6/WID22241/4/ARMENT//SRN_3/NIGRIS_4/3/CANELO_9.1/5/TARRO_1/2*YUAN_1//	1176	665298	700746	*Triticum turgidum*	Spring	CIMMYT
HYPERNO/6/WID22241/4/ARMENT//SRN_3/NIGRIS_4/3/CANELO_9.1/5/TARRO_1/2*YUAN_1//	1177	665298	700747	*Triticum turgidum*	Spring	CIMMYT
HYPERNO/6/WID22241/4/ARMENT//SRN_3/NIGRIS_4/3/CANELO_9.1/5/TARRO_1/2*YUAN_1//	1178	665298	700748	*Triticum turgidum*	Spring	CIMMYT
TJILKURI/11/E90040/MFOWL_13//LOTAIL_6/3/PROZANA/ARLIN//MUSK_6/9/USDA595/3/D67.3/	1179	665306	700749	*Triticum turgidum*	Spring	CIMMYT
TJILKURI/11/E90040/MFOWL_13//LOTAIL_6/3/PROZANA/ARLIN//MUSK_6/9/USDA595/3/D67.3/	1180	665306	700750	*Triticum turgidum*	Spring	CIMMYT
WID802/11/E90040/MFOWL_13//LOTAIL_6/3/PROZANA/ARLIN//MUSK_6/9/USDA595/3/D67.3/	1184	665313	700754	*Triticum turgidum*	Spring	CIMMYT
WID22241/4/ARMENT//SRN_3/NIGRIS_4/3/CANELO_9.1/5/TARRO_1/2*YUAN_1//AJAIA_13/	1189	636383	700765	*Triticum turgidum*	Spring	CIMMYT
WID22241/4/ARMENT//SRN_3/NIGRIS_4/3/CANELO_9.1/5/TARRO_1/2*YUAN_1//AJAIA_13/	1192	636384	700773	*Triticum turgidum*	Spring	CIMMYT
WID22241/4/ARMENT//SRN_3/NIGRIS_4/3/CANELO_9.1/5/TARRO_1/2*YUAN_1//AJAIA_13/	1194	636385	700776	*Triticum turgidum*	Spring	CIMMYT
WID22241/4/ARMENT//SRN_3/NIGRIS_4/3/CANELO_9.1/5/TARRO_1/2*YUAN_1//AJAIA_13/	1195	636385	700778	*Triticum turgidum*	Spring	CIMMYT
WID22241/4/ARMENT//SRN_3/NIGRIS_4/3/CANELO_9.1/5/TARRO_1/2*YUAN_1//AJAIA_13/	1196	636385	700780	*Triticum turgidum*	Spring	CIMMYT
ZENIT/5/SORA/2*PLATA_12//RASCON_37/4/ARMENT//SRN_3/NIGRIS_4/3/CANELO_9.1/6	1202	636394	700794	*Triticum turgidum*	Spring	CIMMYT
ZENIT/5/SORA/2*PLATA_12//RASCON_37/4/ARMENT//SRN_3/NIGRIS_4/3/CANELO_9.1/6	1203	636394	700796	*Triticum turgidum*	Spring	CIMMYT
ZENIT/5/SORA/2*PLATA_12//RASCON_37/4/ARMENT//SRN_3/NIGRIS_4/3/CANELO_9.1/6	1205	636394	700798	*Triticum turgidum*	Spring	CIMMYT
ZENIT/5/SORA/2*PLATA_12//RASCON_37/4/ARMENT//SRN_3/NIGRIS_4/3/CANELO_9.1/	1206	636394	700799	*Triticum turgidum*	Spring	CIMMYT
CBC 509 CHILE/SOMAT_3.1//BOOMER_18/LOTUS_4/6/SOMAT_3/PHAX_1//TILO_1/LOTUS_4/3	1271	636268	700916	*Triticum turgidum*	Spring	CIMMYT
MÂALI/8/GREEN_2/HIMAN_12//SHIP_1/7/ECO/CMH76A.722//BIT/3/ALTAR 84/4/AJAIA_2	1312	636700	700989	*Triticum turgidum*	Spring	CIMMYT
MÂALI/5/1A.1D 5 + 1-06/3*MOJO//RCOL/4/ARMENT//SRN_3/NIGRIS_4/3/CANELO_9.1/11	1336	665421	701030	*Triticum turgidum*	Spring	CIMMYT
STOT//ALTAR 84/ALD/3/PATKA_7/YAZI_1/4/ARMENT//SRN_3/NIGRIS_4/3/CANELO_9.1/5	1359	665531	701067	*Triticum turgidum*	Spring	CIMMYT
RISSA/GAN//POHO_1/3/PLATA_3//CREX/ALLA/11/CANELO_9.1/SNITAN/10/PLATA_10/6	1365	665552	701076	*Triticum turgidum*	Spring	CIMMYT
SOMAT_4/INTER_8/5/AJAIA_16//HORA/JRO/3/GAN/4/ZAR/11/CANELO_9.1/SNITAN/10	1511	636060	701332	*Triticum turgidum*	Spring	CIMMYT
HYPERNO/7/ADAMAR_15//ALBIA_1/ALTAR 84/3/SNITAN/4/SOMAT_4/INTER_8/5/	1564	665435	701396	*Triticum turgidum*	Spring	CIMMYT
JANDAROI/12/ZHONG ZUO/2*GREEN_3//SORA/2*PLATA_12/10/PLATA_10/6/MQUE/4	1566	665437	701398	*Triticum turgidum*	Spring	CIMMYT
TJILKURI/3/GERUFTEL-1//GUAYACAN INIA/2*SNITAN	1623	665235	701476	*Triticum turgidum*	Spring	CIMMYT
TJILKURI/3/GERUFTEL-1//GUAYACAN INIA/2*SNITAN	1624	665235	701477	*Triticum turgidum*	Spring	CIMMYT
WID802/3/TUNSYR-2//GUAYACAN INIA/2*SNITAN	1626	665252	701479	*Triticum turgidum*	Spring	CIMMYT
PBW343 **(Check)**	PBW343	584003	181003	*Triticum aestivum*	Spring	CIMMYT
CACUKE **(Check)**	Cacuke	573163	181016	*Triticum aestivum*	Spring	CIMMYT

Asterix (*) Means backcross.

Single slash (/) Means first cross.

Double slash (//) Means second cross.

Double slash with a number i.e /3/ Means third cross.

### 2.3 Experimental procedure

This study was conducted in a field where canola (*Brassica napus*) was previously grown. A suitable seedbed was prepared using disc plough and harrow operations. Each line was sown in 2-row plots of 0.2 m × 0.75 m. The plots were arranged in 7 × 7 alpha-lattice design with 0.2 m spacing between rows and 0.5 m spacing between blocks. This allowed control of variability within blocks and replications, enhancing the reliability of comparisons among genotypes across environments/seasons. Three replicates were used. At the time of sowing, di-ammonium phosphate (DAP) fertilizer was applied at a rate of 125 kg ha-1 to provide 22.5 kg N and 25.1 kg P. Spreader rows containing susceptible wheat genotypes and *Sr24*-carrying lines were sown between replicates, creating quad rows around the experimental unit. *Sr24*-carrying lines included served as a reference for comparative analysis of genotype susceptibility, and buffering each unit to standardize disease exposure, thus ensuring vigorous assessment of resistance under field conditions. To initiate disease infection, the spreader rows were artificially inoculated with mixture of stem rust urediniospores of different races (TTKSK, TTKST, TTKTT, TTKTK, and TTTTF). Urediniospores harvested from the disease nursery at KALRO-Njoro were suspended in distilled water containing drop of Tween 20 at a concentration of 1×10^5^ spores per ml and were inoculated into the spreader rows using a syringe. The inoculation process was repeated multiple times. Additional, urediniospores were sprayed on the spreader rows to enhance disease development. Overhead irrigation was employed in off-season nurseries to maintain a humid environment conducive to disease development, operating for four hours each during the morning and evening. Pre-emergence herbicide Stomp 455C (pendimethalin) was applied after planting, while post-emergence herbicide Buctril MC (bromoxynil octanoate) and MCPA ethyl-hexyl ester were applied at specific growth stages. Calcium ammonium nitrate (CAN) was top-dressed twice at tillering and stem elongation growth stage to supply nitrogen. Systemic insecticide Thunder OD 145 containing imidacloprid and beta-cyfluthrin was applied at the tillering and ear emergence stages. Foliar diseases were not controlled in this trial. Sprinkler and drip irrigation systems were installed to provide additional moisture during dry periods.

### 2.4 Data collection

The severity of stem rust disease was assessed using the modified Cobb’s scale, which involved determining the percentage of stem area covered by rust pustules (Peterson et al., 1948). Infection response was scored based on pustule size, chlorosis, and necrosis on the stem (Roelfs, 1992). Data collection began when approximately 50% of the test genotypes had headed and susceptible checks displayed 50% disease severity. Stem rust severity was recorded at 7-8-day intervals throughout the study. Response classes included “0” for no visible infection, “R” for resistance, “MR” for moderate resistance, “MS” for moderate susceptibility, and “S” for susceptibility (Peterson et al., 1948). In cases where there was an overlapping infection response on a single genotype, the most frequent response was recorded first, followed by the less frequent one. Stem rust severity was scored three times per season at 7-day intervals. The area under disease progress curve (AUDPC) was calculated to quantify the disease data, and the final severity score was also used in the analysis (Gregory and Finney, 1977).


$$AUDPC=\sum_{i=1}^{n=1}\frac{y_{i}+y_{i+1}}{2}\times (t_{i}+1-t_{i})$$

Where $y_{i}$ =disease observation (severity) at the *i^th^* genotype, $t_{i}$ =time (days) at the *i^th^* disease observation, n=total number of observations.

Phenological traits, such as plant height, spike length, seeds per spike, 1000-kernel weight, and grain yield, were measured for all durum genotypes. Heading was determined when 50% of the spike emerged from the boot. At maturity, plant height was measured from the soil level to the tip of the spikes, excluding awns, in a random sample of 3 plants per plot. Spike length was measured from base to tip, excluding awns, in a random sample of three spikes per plot. The number of seeds per spike was calculated using three random spikes per plot. Grain yield was estimated by harvesting the plots at the base and recorded in kilograms per plot area (Kg m^−2^), then converted to t ha^−1^ as:


$$\text{GY }(\text{t }{\text{ha}}^{-1})\frac{10,000\;m^{2}}{PAm^{2}}\times \frac{GY\;plot^{-1}(Kg)}{10000\;Kg}$$

where; GY=grain yield and PA=plot area.

Using a Contador seed counter (brand *Pfeuffer*, Serial number: 14176107), 1,000 kernels were counted from threshed grains and weighed to estimate 1000-Kernel weight.

### 2.5 Statistical analysis

Statistical analyses were performed to evaluate the significance of seasonal and genotypic effects on disease severity and agronomic traits. The genotype by environment (GxE) interactions were analyzed using GEA-R (Genotype x Environment Analysis with R), which allows for the visualization and interpretation of GxE interactions (Pacheco-Gil et al., 2015). Broad-sense heritability estimates were calculated to determine the genetic influence on these traits. Negative correlations between disease parameters and agronomic traits were identified to explore potential trade-offs between resistance and agronomic performance.

### 2.6 Data analysis

The disease and agronomic data underwent two-step combined analysis of variance. First, individual seasons were analyzed, and then combined analysis was performed using 7 × 7 alpha-lattice design. The general linear model procedure (GLM) of SAS version 9.4 (SAS Institute, N.C, 2009) was used for the analysis.


$$Y_{ijklm}=\mu +S_{i}+R_{j(i)}+B_{k(ji)}+\;G_{l}+\;GS_{li}+\;\epsilon_{ijklm}$$

where *Y _ijkl_* =observation of the experimental units; *µ* =overall mean; S*_i_* =effects due to *i^th^* season; *R_j(i)_* =effects due to the *j^th^* replicate nested in the *i^th^* season; *B _k(ji) _* =effects due to the *k^th^* block nested in the *j^th^* replicate and the *i^th^* season; *G_l_* = effects due to *l^th^* genotype in the *i^th^* season; *GS_li_* = effects due to the interaction of the *j^th^* genotype and *i^th^* season and *ε_ijklm_* = residual.

Tukey’s honestly significant difference (Tukey, 1949) was used to compare means of the agronomic and disease traits. The mixed model procedure of SAS was employed to analyze both the disease and agronomic data, allowing for correction of experimental effects such as seasons, replicates, and blocks variability. The mixed model estimated the random effects of experiment, replicates, blocks, and seasons variables, which were then removed from the trait values of each genotype. This process resulted in Best Linear Unbiased Predictors (BLUPs) or adjusted means (LSMeans) for each genotype. The mean BLUPs from all seasons were used for subsequent analyses, as they provide estimates of genotype effects that closely align with true genotypic effects. Broad-sense heritability was estimated based on genotype mean values.


$$H^{2}=\frac{\sigma_{g}^{2}}{\sigma_{g}^{2}+\frac{\sigma_{gs}^{2}}{s}+\frac{\sigma_{e}^{2}}{sr}}$$

where, $\sigma_{g}^{2}$ =genotypic variance, r=number of replicates, s=number of seasons, $\sigma_{gs}^{2}$ =genotype × season interaction variance and $\sigma_{e}^{2}$ =residual variance.

The GGE biplot, utilizing principal components scores from singular value partitioning (SVP), was employed to depict the impact of genotype and genotype by environment interaction on stem rust severity using GEA-R Software (Angela et al., 2015). The average environment coordination (AEA) from the biplots assisted in identifying the top-performing genotypes and selecting stable and high-performing genotypes. Polygons were utilized to determine the best-performing genotypes in each season. The GGE model is given by;


$$Y_{ij}=\mu +\;E_{j}+\;\sum_{k=1}^{k}b_{ik}z_{jk}+\;\epsilon_{ij}$$

where, $Y_{ij}$ =the mean performance of *i^th^* genotype in *j^th^* environment, μ = the grand mean, $E_{j}$ = main effect of *j^th^* environment, k=multiplicative term for genotype by environment interaction, $b_{ik}$ =genotypic score, $z_{jk}$ =environmental score, $\epsilon_{ij}$ =error term.

Correlations among the traits evaluated were computed from the BLUPs to establish the association among all the agronomic and disease traits. The correlation coefficients were further used to determine direct and indirect effects of each disease trait to agronomic traits. The principal component analysis (PCA) was conducted using GENSTAT (Version 16), correlation matrix to determine the relationship between durum traits collected in this study. The PCA allows explanation of the effect of multiple traits that are acting in almost similar ways.

## 3 Results

### 3.1 Combined analysis of variance

The combined analysis of variance across seasons demonstrated significant effects of seasons on stem rust severity (p ≤ 0.001) and area under disease progress curve (AUDPC) (p ≤ 0.01), as well as on various agronomic traits (**Table 2**). Genotypes also exhibited significant effects (p ≤ 0.001) on both disease parameters and agronomic traits (**Table 2**). Furthermore, significant interaction (p ≤ 0.001) between genotype and season was observed for stem rust severity, AUDPC, and spike length, indicating genotypic performance across different seasons (**Table 2**).

**Table 2:** Combined analysis of variance for stem rust severity and agronomic traits on 49 durum genotypes evaluated for 3 seasons under stem rust epiphytotic conditions.

Source of variation	df	FDS	AUDPC	SPL	PH	KPS	KW	Yield
Season (S)	2	15240.22***	583817.86**	320291.29***	14493.57***	8305.59***	2492.85***	155.21**
Replicate (S)	6	552.88	139737.58	145.37	335.99	137.70	187.03	6.46
Block (S × R)	54	99.58	186262.69	46.44	88.61	96.76	32.33	1.35
Genotype (G)	48	2202.71***	3524160.19***	173.35***	241.77***	175.29***	77.61***	5.07***
G × S	94	375.82***	737125.03***	71.51*	54.93	95.75451	20.44	1.55***
Error		236	1858.60	52.72	65.37	76.83	21.51	0.29
CV (%)		19.21	29.22	11.73	9.68	19.44	18.90	18.55
*R^2^*		0.95	0.93	0.98	0.78	0.70	0.76	0.93

*, **, and ***, significant at p ≤ 0.05, p ≤ 0.01, and p ≤ 0.001, respectively, df, degree of freedom; FDS, Final disease severity; AUDPC, area under disease progress curve; SPKL, spike length; PH, plant height; KPS, kernels per spike; TKW, 1000-kernel weight.

The three test seasons significantly influenced the performance of genotypes in terms of agronomic traits and the occurrence and progression of the disease. Season 1 exhibited higher stem rust severity compared to seasons 2 and 3 (**Table 3**), with disease progression also more rapid in Season 1 than in other seasons. The higher disease pressure in Season 1, likely due to more favorable environmental conditions for pathogen development such as higher humidity, resulted in stressed plants reallocating resources towards defense rather than growth, thereby limiting spike length (SPL).

**Table 3:** Effect of seasons on stem rust severity, Area under disease progress curve, spike length, plant height, kernels per spike, 1000-kernel weight and yield of durum genotypes.

Season	Severity	AUDPC	SPL	PH	KPS	TKW	Yield
1	45.48a	198.71a	7.76c	72.65c	42.17b	24.34ab	2.35b
2	25.79b	116.70b	85.93b	85.93a	39.21b	20.42b	2.24b
3	31.73b	127.08b	91.97a	91.97a	53.86a	28.88a	4.09a
Tukey MSD _(0.05)_	8.42	54.62	4.32	6.56	4.20	4.89	0.91

Means followed by the same letters are not significantly different at p ≤ 0.05. AUDPC, Area under disease progress curve; SPKL, spike length; PH, plant height; KPS, kernels per spike; TKW, 1000-kernel weight.

Season 3 resulted in the longest spikes, followed by season 2 and season 1. Plant growth was similar in seasons 1 and 3, but the growth rate was limited in season 1 (**Table 3**), suggesting that the environmental stresses affecting spike development did not similarly impact overall plant growth rate. Season 3 favored kernels per spike (KPS) and yield, which were comparable to those in season 1 and season 2. Additionally, the thousand kernel weight (TKW) increased from season 2 to season 3. Although disease incidence was lower in Season 2, kernel weight remained reduced, likely affected by suboptimal abiotic conditions such as transient drought or heat stress during the crucial grain-filling period, which adversely impacted kernel development.

### 3.2 Broad-sense heritability (H^2^) estimates

Broad sense heritability varied across traits, ranging from 0.90 for AUDPC to 0.35 for KPS, indicating the extent to which genetic factors influenced disease and agronomic traits over environmental factors (**Table 4**). Traits such as final disease score, AUDPC, spike length, plant height, kernel weight, and yield showed high heritability estimates (**Table 4**). However, KPS exhibited the lowest heritability estimate of 0.35, suggesting stronger influence of environmental effects on this trait. The environmental variance for KPS was nine times greater than the genotypic variance, while the residual variance was six times greater than the genotypic variance (**Table 4**).

**Table 4:** Heritability, Variance components, and means of stem rust severity, area under disease progress curve and yield and yield components of durum genotypes evaluated over three seasons.

Statistic	FDS	AUDPC	SPL	PH	KPS	KW	Yield
Heritability (*H^2^*)	0.84	0.91	0.89	0.80	0.35	0.81	0.76
$\sigma_{g}^{2}$	237.79	28637.44	1.30	37.03	6.23	9.48	0.52
$\sigma_{genv}^{2}$	108.26	6347.36	0.00	0.00	23.49	0.00	0.25
$\sigma_{env}^{2}$	100.37	5814.79	0.29	16.88	55.34	16.47	0.98
$\sigma_{E}^{2}$	63.80	7789.48	1.39	55.31	35.90	21.02	0.73

AUDPC, area under disease progress curve; SPKL, spike length; PH, plant height; KPS, kernels per spike; TKW, 1000-kernel weight.

### 3.3 Correlation analysis among agronomic and disease traits

At the phenotypic level, both stem rust severity and AUDPC exhibited significant negative correlations with all agronomic traits (**Table 5**). The significant negative correlations suggest an association between stem rust severity, AUDPC, and agronomic traits in the overall phenotype. Nevertheless, it is worth mentioning that the phenotypic correlation between spike length (rp=-0.23), plant height (rp=-0.26), and AUDPC was not statistically significant (**Table 5**). This implies that spike length and plant height may not have a substantial direct impact on the severity or progression of stem rust.

**Table 5:** Phenotypic (bold) and genotypic correlation of final disease score, area under disease progress curve and agronomic traits of durum traits bold=phenotypic correlation.

Traits	Final disease score	AUDPC	SPL	PH	KPS	KW
AUDPC	0.99***					
	0.98***					
SPL	-0.49***	-0.40**				
	-0.31*	**-0.23**				
PH	-0.46***	-0.37**	1.00***			
	0.34*	**-0.26**	0.91***			
KPS	-0.49***	-0.66***	0.98***	0.63***		
	-0.45**	-0.50***	0.35*	0.30*		
KW	-0.58***	-0.58***	1.00***	0.87***	0.44**	
	-0.52***	0.51***	0.63***	0.68***	0.51***	
Yield	-0.47***	-0.49***	0.95***	0.88***	0.63***	0.83***
	-0.41**	-0.41**	0.55***	0.63***	0.58***	0.81***

*, **, and ***, significant at p ≤ 0.05, p ≤ 0.01, and p ≤ 0.001, respectively, AUDPC, Area under disease progress curve; SPKL, spike length; PH, plant height; KPS, kernels per spike; TKW, 1000-kernel weight.

### 3.4 Effects of genotypes, environments, and genotype × environment interactions of stem rust severity and yield

**Figure 3:**
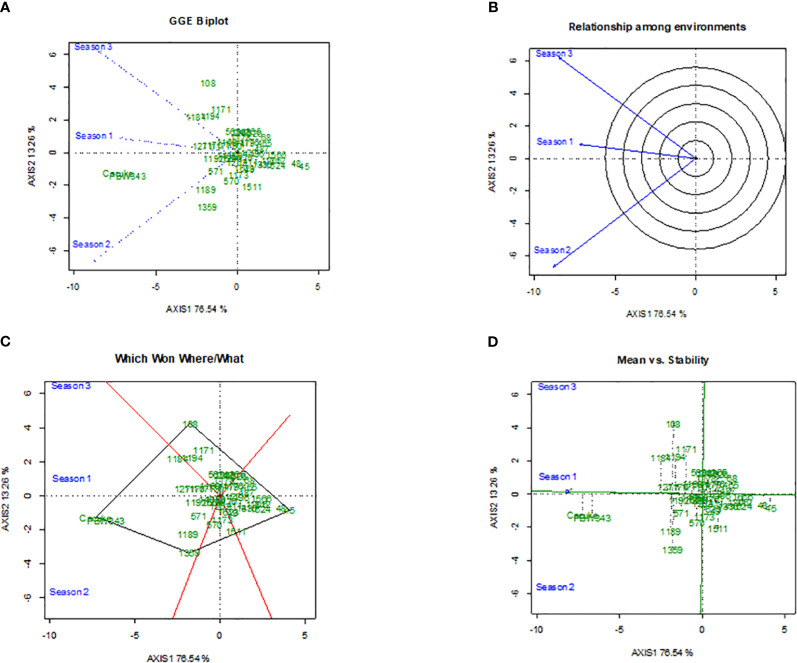
The Genotype main effect and Genotype × Environment interaction biplots depicts the performance of 49 durum wheat genotypes in terms of stem rust severity across three seasons,with subfigures showing the GGE biplot for main effects and interactions **(A)**, the radial relationship among environments **(B)**, polygon view highlighting top performers by environment **(C)**, and plot of mean performance versus stability **(D)**.

**Figure 4:**
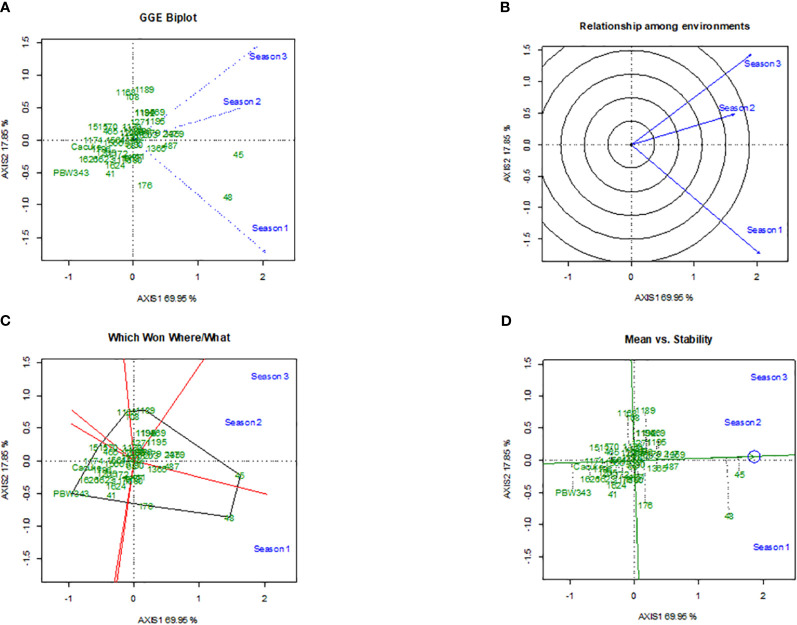
The Genotype main effect and Genotype × Environment interaction biplots illustrates the stability of 49 durum wheat genotypes’ grain yield across three seasons,featuring GGE biplot for relationship and discrimination by seasons **(A)**, radial plot of the relationship among environments **(B)**, polygon view identifying top-performing genotypes in each environment **(C)**, and plot assessing mean yield versus stability **(D)**.

The GGE biplots for stem rust severity (**Figure 3**) and yield (**Figure 4**) provided comprehensive analysis of durum wheat genotypes’ performance across different seasons. The disease biplot explained 89.80% of the variation, with PC1 accounting for 76.54% and PC2 for 13.26%, while the yield biplot explained 87.80% of the variation, with PC1 at 69.95% and PC2 at 17.85%. Genotype 45 stood out in both biplots, showing strong resistance to stem rust, particularly in Season 2, and high yield performance, though with variable stability. Genotype 48 also demonstrated strong performance, especially in Season 1, with better stability in both disease resistance and yield (**Figures 3A**, **4A**). Genotypes 108, 1171, and 1184 showed significant interactions with environmental factors, indicating distinct responses that could be leveraged for targeted breeding. **Figures 3B** and **4B** highlight the similarity between Seasons 1 and 3, suggesting that genotypes were performing well in Season 1 were likely to do similarly in Season 3, while Season 2 differed significantly, indicating that genotypes might respond differently due to unique environmental factors like temperature and humidity. **Figures 3C** and **4C** identified top performers in specific environments: Genotype 45 excelled in Season 2, and Genotype 48 excelled in Season 1. Other genotypes, such as 108 and 1168, also showed good performance under certain conditions, highlighting their adaptability. **Figures 3D** and **4D** assess mean performance against stability, with Genotype 45 showing high performance but variable stability, and Genotype 48 exhibiting high performance with better stability. Genotypes 108, 1168, and 1189, positioned closer to the origin, demonstrate average performance with high stability, making them reliable across different environments.

### 3.5 Principal component analysis of durum wheat traits

**Figure 5:**
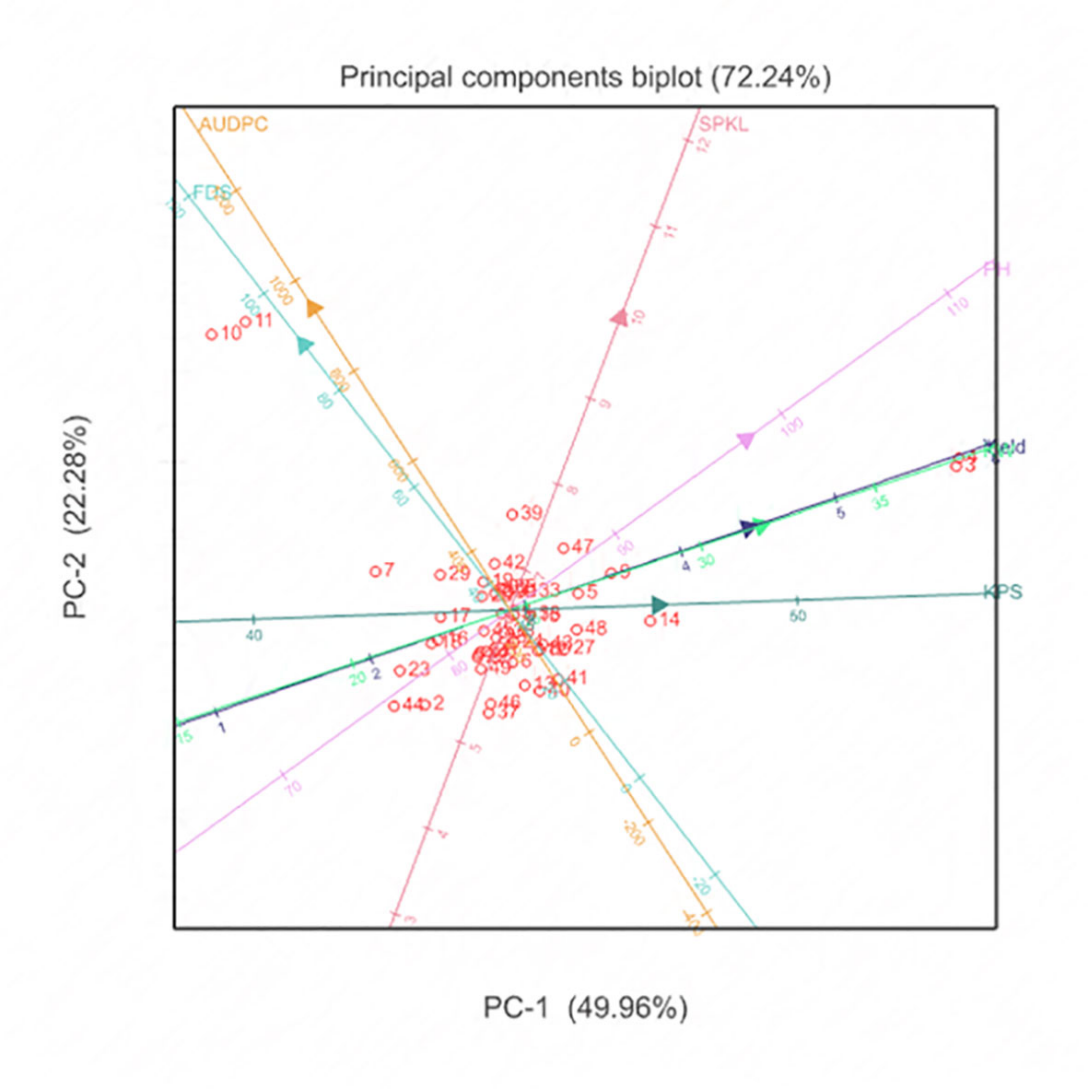
Principal Component Analysis show performance similarities among the evaluated 49 durum genotype traits through multiple trait analysis.

The Principal Component Analysis (PCA) biplot, was used to capture the performance similarities among genotypes based on multiple trait analyses. This biplot explained 72.24% of the total variance, with Principal Component 1 (PC1) accounting for 49.96% and Principal Component 2 (PC2) accounting for 22.28% (**Figure 5**). Genotypes with high yield were strongly differentiated along PC1, indicating that yield was a major determinant of overall performance. Traits associated with PC2, such as lower Area Under Disease Progress Curve (AUDPC) values, highlighted disease resistance as key for genotype differentiation. Genotypes exhibiting high yield and plant height were identified as key candidates for high-yield breeding programs, while those with lower AUDPC values were vital for breeding disease-resistant varieties. Most genotypes clustered around the origin, showing balanced performance and stability across multiple traits, suggesting reliability under diverse environments.

## 4 Discussion

This study found significant seasonal variations in response of durum wheat genotypes to stem rust infections, confirming the substantial phenotypic diversity among the genotypes tested. These variations highlighted the role of environmental factors in expression of stem rust severity, as measured by FDS and AUDPC. The study’s ability to capture these variations is emphasized by our rigorous approach and extensive evaluation conducted over several seasons. Similarly, findings of Shaw et al. (2008) and Mideksa et al. (2018), noted that environmental conditions, such as rainfall and temperature, play role in progression of stem rust, with temporal factors having significant impact on disease dynamics. However, our focus on specific geographical locations might limit the generalizability of these results to other regions experiencing different climatic conditions. Furthermore, our findings that seasonal conditions contribute to variations in disease severity emphasized the complex influence of environmental factors, as noted by Nopsa and Pfender’s (2014) observation that environmental conditions can sometimes overshadow genetic resistance. Other insights also from Sgrò and Hoffmann (2004), highlighted the significant impact of environmental variations on phenotypic trait expression and genetic correlations. Season 1 exhibited significantly greater disease severity compared to Seasons 2 and 3, suggesting that the environmental conditions during this period exacerbated disease symptoms. This also aligns with Velásquez et al.’s (2018) findings, which demonstrated how weather conditions intricately affect plant-pathogen interactions.

A significant negative correlation was found between area under the disease progression curve (AUDPC), final disease severity (FDS), and yield components. These findings highlight the importance of effective resistance genes in maintaining high yields even in the face of disease, demonstrating the impact of genetic resistance in agricultural settings. Other studies, Ogutu and Charimbu (2020); Msundi et al. (2021) and Wanyoike et al., 2022 also discovered that both AUDPC and FDS had negative impact on grain yield, emphasizing the importance of genetic resistance in mitigating disease-induced yield reductions.

The race-specific gene *Sr13* has been essential in providing resistance against *Ug99* race of stem rust and its variants in durum wheat (Singh et al., 2015). Despite the effectiveness of *Sr13* (Zhang et al., 2017), the emergence of non-*Ug99* races such as TTKSK, JRCQC, and TTRTF, which have developed virulence against alleles like *Sr13b* and *Sr9e* (Olivera et al., 2012), underscores the dynamic nature of pathogen evolution and the challenges it poses (Olivera et al., 2019). These races have developed virulence against alleles like *Sr13b* and *Sr9e*, which differ from *Sr13* primarily in their genetic makeup and the resulting functional responses to the pathogen. For instance, *Sr13b* might confer resistance through slightly altered protein configuration that affects its interaction with the pathogen, leading to differential susceptibility when compared to *Sr13*. The presence of *Sr13* and related resistance alleles in the genetic backgrounds of durum wheat genotypes we assessed is crucial, as it continuously necessitates updates in breeding strategies to effectively counter these evolving threats. Mergessa et al. (2020) drew attention to the necessity of constant innovation in breeding strategies, highlighting the implications of our findings for continued adaptability in the face of changing disease threats.

Our findings revealed that approximately 40.82% of durum wheat genotypes, exhibiting up to 30% stem rust severity, were categorized as resistant to moderately resistant. These partially resistant genotypes underscore the effectiveness of *Sr* genes, under field conditions. However, to fully utilize *Sr* gene-mediated resistance in breeding programs, additional research into the genetic backgrounds and environmental interactions is likely necessary given the heterogeneity in resistance levels between genotypes. This resistance is crucial not only for maintaining yield but also for reducing the selection pressure that drives the evolution of virulence in pathogen populations. However, the variability in resistance levels across genotypes suggests further exploration into the genetic backgrounds and environmental interactions is necessary to fully leverage *Sr* gene-mediated resistance in breeding programs. Studies by Olivera et al. (2021) also demonstrated similar levels of resistance in USDA durum wheat accessions, noting that partial resistance can effectively slow the adaptation of pathogens by reducing selection pressure for virulent mutations. Research by Burdon and Laine (2019) on the evolutionary dynamics of plant-pathogen interactions suggested that partial resistance, involving spectrum of resistance genes, may provide more sustainable approach to managing disease than relying on complete resistance from single genes. These models propose that such strategies not only mitigate the risk of resistance breakdown but also contribute to maintaining crop yield under various stress conditions.

Our research showed genetic influences on key traits such as plant height and final disease score, with broad-sense heritability estimates indicating strong genetic basis. However, the low heritability of kernels per spike (KPS) suggested significant environmental impact, complicating selection based solely on this trait. Furthermore, we found negative correlations between disease severity (AUDPC) and key agronomic traits like KPS, kernel weight, and grain yield. These correlations, as also described by Chiko et al. (2022), imply that lower disease severity and increased biomass are linked to higher yields and grain weight. Our findings agree with those of Willocquet et al. (2018, 2022), who found that increased disease severity not only reduces plant height, but also redirects resources from reproductive structures to pathogen spore growth during critical growth phases. This redirection has significant impact on grain quality and yield, highlighting the importance of selecting for traits that improve resource allocation to reproductive structures in order to reduce stem rust impacts and maintain yield under disease pressure. Such physiological impacts suggest that breeding for traits that enhance resource allocation to reproductive structures could mitigate the effects of stem rust, thus preserving yield under disease pressure.

Durum wheat production in Kenya is relatively low compared to other countries, such as Ethiopia (Tidiane Sall et al., 2019). Therefore, choosing high-yielding, stable genotypes for use in breeding programs is essential to enhance durum wheat productivity. A comprehensive understanding of genotype-by-environment interactions (GEI) is crucial for setting breeding goals, selecting optimal testing locations, and making variety recommendations (Yan and Hunt, 2001). This understanding is also vital for assessing the adaptability and stability of different genotypes. Our research showed that the major source of variability came from G × E interactions, which suggested significant environmental influence on genotype performance. This implies that genotypes developed for specific environments might not perform consistently well in other settings, highlighting the need to broaden the scope of evaluation trials across various ecological conditions. Additionally, it’s important to cultivate identified genotypes in their ideal environments to maximize the benefits of positive GEI (Khan et al., 2021). Thus, identifying suitable environments for breeding and choosing strategic sites for genotype testing are key priorities.

In our study, we used the GGE biplot method to evaluate the interaction of three environments with genotype performance, focusing on stem rust severity and grain yield. The analysis pinpointed genotypes that were stable and specifically adapted to different environmental conditions, which is vital for effective breeding strategies. The results underscore a crucial link between yield stability and disease resistance in the evaluated genotypes. For example, Genotype 45 displayed strong resistance to stem rust in the second season and also showed high yield performance, suggesting that breeding for disease resistance does not necessarily detract from yield potential. By selecting for both traits, robust varieties suitable for various conditions can be developed. Genotype 48 was notable for its stability in both disease resistance and yield, reinforcing the potential for breeding high-performing, disease-resistant wheat. The significant GxE interactions observed highlight the importance of testing genotypes in multiple settings. These interactions reveal the unique performance of specific genotypes, like 1171 and 1184, under varying environmental conditions, providing essential insights for breeders in selecting adaptable and resilient varieties. Furthermore, the observed genotype × environment interactions emphasize the need for breeding programs to consider both resistance and yield stability. This reflects the complex interplay of genetics and environmental influences, further complicated by shifting climatic conditions that can exacerbate disease severity and affect water availability. Hence, developing genotypes that are not only resistant to pathogens but also resilient to abiotic stresses such as drought and heat is critical (Ramegowda and Senthil-Kumar, 2015; Pandey et al., 2017). This approach is essential, particularly in areas prone to extreme weather events, highlighting the need for multi-environment trials to capture the full performance potential of genotypes, as supported by other findings from Tekdal and Kendal (2018) and Madahana et al. (2021).

Principal component analysis (PCA) on the other hand, is a multivariate technique that helps to understand the interrelationships among multiple traits by transforming them into a set of new, uncorrelated variables called principal components (Mishra et al., 2017; Greenacre et al., 2022). This technique is particularly valuable in plant breeding as it enables the identification of key traits contributing to genetic diversity and performance across different environments. PCA provides visual representation of the patterns of similarity among genotypes, allowing breeders to pinpoint those with desirable characteristics, such as high yield and disease resistance, while maintaining stability under various environmental conditions. Here, PCA offered insights into the performance similarities among durum wheat genotypes based on multiple trait analyses. The PCA biplot explained 72.24% of the total variance, with Principal Component 1 (PC1) accounting for 49.96% and Principal Component 2 (PC2) for 22.28%. This analysis differentiated genotypes based on key traits, emphasizing the importance of yield and disease resistance. For instance, genotypes with high yield were strongly associated with PC1, highlighting yield as a major determinant of overall performance. On the other hand, traits related to PC2, such as lower Area Under Disease Progress Curve (AUDPC) values, highlighted the importance of disease resistance in genotype differentiation. The clustering of genotypes around the origin indicated balanced performance and stability across multiple traits, suggesting their reliability under diverse environmental conditions. This aligns with the findings of Alemu et al. (2020), who emphasized the importance of genetic variability and trait associations in Ethiopian durum wheat landraces.

## 5 Conclusion

This research conducted comprehensive analysis of genotype-environment interactions impacting durum wheat in Kenya, shedding light on the trade-offs between stem rust resistance and agronomic performance. Our findings identified specific genotypes such as G45 and G48, that consistently demonstrate resistance to stem rust across various environmental conditions, marking them as ideal candidates for breeding programs aimed at enhancing crop resilience and productivity. The high resistance to stem rust ensures lower disease incidence and severity, which translates to healthier plants and higher yields. Moreover, their stable performance across diverse environments implied robust adaptability, crucial for resilience against climatic variability. Additionally, the application of genotype and genotype by environment (GGE) biplot analysis had been instrumental in identifying these genotypes that not only perform well agronomically but also withstand the pressure of stem rust. To enhance food security and promote sustainable agriculture in Kenya and potentially in similar regions globally, it is essential to focus on breeding programs that balance yield stability and disease resistance. Leveraging the genetic diversity within durum wheat will enable breeders to develop varieties tailored to Kenya’s diverse agro-ecological zones, ultimately ensuring robust and resilient crop performance across varying conditions.

## Data availability statement

The original contributions presented in the study are included in the article/supplementary material. Further inquiries can be directed to the corresponding authors.

## Author contributions

EO: Conceptualization, Methodology, Writing – original draft, Writing – review & editing. SM: Data Curation, Formal analysis, Writing – review & editing. SB: Investigation, Resources, Writing – review & editing. GM: Supervision, Writing – review & editing.
